# Post-traumatic stress in healthcare workers during the COVID-19 pandemic: a cross-sectional study

**DOI:** 10.1192/j.eurpsy.2024.1384

**Published:** 2024-08-27

**Authors:** Z. Athimni, N. Belhadj Chabbeh, S. Chatti, F. Chelly, L. Ben Afia, M. Bouhoula, A. Chouchane, A. Aloui, I. Kacem, M. Maoua, A. Brahem, H. Kalboussi, O. El Maalel

**Affiliations:** ^1^Occupational Medicine and Professional Pathologies Department, Farhat Hached Teaching Hospital, Avenue Ibn El Jazzar, Sousse, 4000, Tunisia; ^2^Occupational Medicine and Professional Pathologies Department, Sahloul Teaching Hospital, Tunisia, Sousse, Tunisia

## Abstract

**Introduction:**

During the COVID-19 pandemic, healthcare professionals worked under critical care conditions and had to adapt quickly to extreme work situations. They were confronted with several occupational stressors.

**Objectives:**

To determine the prevalence and factors associated with post-traumatic stress symptoms among healthcare personnel at Farhat Hached Hospital in Sousse during the COVID-19 pandemic.

**Methods:**

This was a descriptive cross-sectional study conducted among care staff at the Farhat Hached University Hospital in Sousse over a 3-month period during the 4th wave of COVID-19. Data were collected using a questionnaire covering socio-professional and medical data. Post-traumatic stress symptoms were assessed using the Impact of Event Scale-Revised (IES-R). Statistical analysis was performed using SPSS.23 software.

**Results:**

Our study included 326 health professionals from the CHU Farhat Hached. The mean age of our population was 36.38 ±10.19 years. The sex ratio was 0.23. Most healthcare staff were married (61.3%) and had dependent children (60.4%). Nurses were the most represented at 32.2%, followed by health technicians (22.7%) and medical residents (18.4%). Average job tenure was 10.62±10.69 years, with extremes ranging from 1 to 39 years. The prevalence of post-traumatic stress disorder was 32.5%. Paramedics were more likely to develop post-traumatic stress symptoms (OR=2.3 (IC95%: 1.4-3.8), p=0.001). Leisure activities were protective factors against post-traumatic stress symptoms (OR=0.4 (IC95%: 0.2-0.8), p=0.018). The multivariate analytical study revealed that being a paramedic and having a personal history of COVID19 infection were independently associated with post-traumatic stress symptoms.

**Conclusions:**

Our results demonstrated the significant impact of the COVID-19 pandemic on the mental health of healthcare personnel. Lessons learned from this pandemic should help in the development of context-specific strategies to support healthcare workers and promote the protection of their mental health.

**Disclosure of Interest:**

None Declared

